# Experimental Evaluation of the Effect of Degradation on the Mechanical Behavior and Morphometric Characteristics of Functionally Graded Polymer Scaffolds

**DOI:** 10.3390/polym16243474

**Published:** 2024-12-12

**Authors:** Nataliya Elenskaya, Ilia Vindokurov, Evgeniy Sadyrin, Andrey Nikolaev, Mikhail Tashkinov

**Affiliations:** 1 Laboratory of Mechanics of Biocompatible Materials and Devices, Perm National Research Polytechnic University, 614990 Perm, Russia; 2 Laboratory for Mechanics of Biomaterials, Don State Technical University, 344000 Rostov-on-Don, Russia

**Keywords:** polymers, degradation, scaffolds, additive technologies, mechanical properties, microcomputed tomography, morphometric analysis, triply periodic minimal surfaces

## Abstract

Bone transplantation ranks second worldwide among tissue prosthesis surgeries. Currently, one of the most promising approaches is regenerative medicine, which involves tissue engineering based on polymer scaffolds with biodegradable properties. Once implanted, scaffolds interact directly with the surrounding tissues and in a fairly aggressive environment, which causes biodegradation of the scaffold material. The aim of this work is to experimentally investigate the changes in the effective mechanical properties of polylactide scaffolds manufactured using additive technologies. The mechanism and the rate of the degradation process depend on the chosen material, contact area, microstructural features, and overall architecture of sample. To assess the influence of each of these factors, solid samples with different dimensions and layers orientation as well as prototypes of functionally graded scaffolds were studied. The research methodology includes the assessment of changes in the mechanical properties of the samples, as well as their structural characteristics. Changes in the mechanical properties were measured in compression tests. Microcomputed tomography (micro-CT) studies were conducted to evaluate changes in the microstructure of scaffold prototypes. Changes caused by surface erosion and their impact on degradation were assessed using morphometric analysis. Nonlinear changes in mechanical properties were observed for both solid samples and lattice graded scaffold prototypes depending on the duration of immersion in NaCl solution and exposure to different temperatures. At the temperature of 37 °C, the decrease in the elastic modulus of solid specimens was no more than 16%, while for the lattice scaffolds, it was only 4%. For expedited degradation during a higher temperature of 45 °C, these ratios were 47% and 16%, respectively. The decrease in compressive strength was no more than 32% for solid specimens and 17% for scaffolds. The results of this study may be useful for the development of optimal scaffolds considering the impact of the degradation process on their structural integrity.

## 1. Introduction

Additive technologies open new opportunities for the creation of geometrically complex objects with optimal mechanical properties. A relevant application for such technologies is overcoming the challenges of personalized biomedicine, particularly in the fields of tissue engineering and surgery. For instance, bone tissue damage requires the use of biocompatible implants to restore integrity, morphology, and, consequently, the original mechanical properties of the patient’s bone [1]. Bone defects, tumor removal, or severe osteoarthritis of joints may necessitate surgical intervention. Joint replacement can aim at regenerating damaged tissues [2,3,4] or completely replacing the affected area [5,6]. It is important that the implant remains stable during its long-term contact with surrounding tissues thus not provoking any pathological reactions. Porous structures (scaffolds) used in tissue engineering facilitate the regeneration process of damaged or removed bone tissues. For successful use, it is essential that the rate of scaffold degradation is comparable to the rate of bone tissue formation. This will ensure the stability of the mechanical properties of the “implant-tissue” system throughout the entire rehabilitation process. Gradual dissolution of the implants while damaged tissues recover eliminates the need for additional surgical intervention when structural support is no longer necessary. The application of such materials is mostly in demand in orthopedics, traumatology, surgery, dentistry and neuro-oncology.

When selecting scaffold materials for bone engineering, the most crucial criterion is biocompatibility, which ensures harmonious interaction with living tissues without causing toxic, inflammatory, or immune reactions [7]. Another important criterion is the rate of biodegradation, which should correspond to the gradual replacement of scaffolds by the body’s cells over time [8]. Biocompatible materials used in tissue engineering include metals, ceramics, polymers, and hydrogels [9,10]. However, metallic implants are rarely biodegradable, and the degradation rate of ceramic implants does not match the rate of bone tissue ingrowth. Among the most promising materials are polymeric materials, which not only meet the requirements for mechanical properties, but also possess necessary dissolution and biocompatibility characteristics while enhancing cell adhesion due to surface roughness [9].

The degradation processes in polymeric scaffolds depend on both the biochemical properties of the material and the internal architecture of the scaffold, its morphometric characteristics, and the loading conditions [11,12]. During this process, the structure of the scaffold changes, altering the size and shape of the pores, which positively affects the formation of cellular tissue. Additionally, drug delivery occurs in a controlled manner due to the rate of degradation [13,14]. At the same time, the rate of this process significantly depends on the individual characteristics of each patient, ranging from the specific features of the cellular matrix to patient’s lifestyle factors [15,16,17].

**Figure 1:**
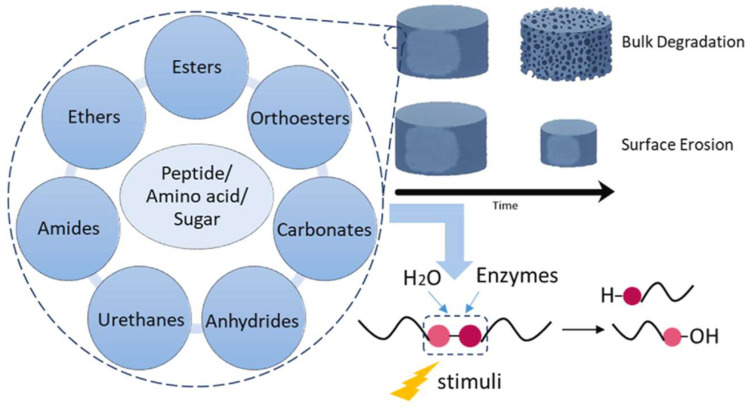
Bulk degradation and surface erosion processes [18].

Degradation of polymer scaffolds occurs mainly due to the hydrolysis process, which depends on several factors. Two distinct degradation mechanisms are distinguished: bulk degradation and surface erosion. Bulk degradation occurs when a polymer loses mass so that monomer diffusion occurs uniformly throughout its volume, while surface erosion occurs when solid materials lose mass from the surface of the structure and then their volume decreases toward the center of the body (Figure 1).

Bulk degradation predominates if the macromolecular chains of the polymer degrade slower than the liquid is absorbed into the polymer, otherwise surface erosion occurs [12]. In this case, bulk degradation and surface erosion represent two extreme cases: as a rule, both types of degradation occur simultaneously, affecting the overall process to different degrees. The underlying degradation mechanism depends on the type of materials. Moreover, the use of additive technologies for manufacturing of such devices leads to the formation of internal defects and the predominance of the bulk degradation mechanism [17,19].

The morphology of the scaffold has a significant influence on the degradation rate: the interconnectedness of pores promotes the circulation of biological fluids, resorption and tissue sprouting, and sufficient specific surface area improves the interaction with surrounding fluids and tissues [20]. However, the specific surface area (SSA, ratio of internal surface area to volume of the structure, mm^−1^) will also significantly affect the degradation rate of the scaffold: a larger area will promote faster degradation and faster implant failure [11,21]. The autocatalytic effect caused by the accumulation of degradation products in the case of small pore size and large wall thickness may also contribute to the acceleration of the degradation process [22,23,24].

Despite the number of previously conducted studies addressing the problems of simulation and experimental investigation of the degradation process of lattice structures, at the moment, there are no reliable models or mechanisms that can predict how the degradation of a structure with a complex heterogeneous architecture will occur. The relevance of this question is connected, firstly, with the fact that additively manufactured implants, on the one hand, allow to take into account individual features of a particular patient, and on the other hand, their complex architecture and manufacturing defects do not allow to predict their behavior under the influence of aggressive (biological) media. In addition, the question of changes in the mechanical properties of the structure at the initial stage of exposure to aggressive media is of interest. In order to answer these questions, the following experimental studies are proposed within the framework of this work:

(i) Analysis of changes in mechanical properties of solid polymer samples to assess the influence of aggressive external environment at early stages of exposure;
(ii) Analysis of the influence of technological parameters of additive manufacturing on the change in the mechanical characteristics of the material under the influence of the external environment;
(iii) Investigation of the influence of liquid medium on additively manufactured scaffolds.

The objects of study are solid specimens and functionally graded structures based on triply periodic minimal surfaces (TPMS) designed for the replacement of damaged bone tissue. Both their mechanical response before and after exposure to a liquid medium and their architectural features caused by defects that arose in the manufacturing process as well as the changes in the degradation process are evaluated.

## 2. Materials and Methods

### 2.1. Design of Solid Samples

The mechanical properties of additively manufactured objects can be significantly influenced by the scale of the structure, printing orientation and its accuracy. Therefore, for preliminary evaluation of the material behavior, standard solid cylindrical specimens were fabricated to evaluate the effect of the scale of sample on the rate and process of degradation of the mechanical properties (Table 1). The material used was the biocompatible and biodegradable polymer polylactide (PLA) (produced by REC, Moscow, Russia) approved for the biomedical applications [25].

The second group of specimens were solid prismatic specimens with vertical and horizontal printing orientation relative to the applied load. They were fabricated to evaluate the effect of the layers’ orientation on the mechanical response during testing (Table 2).

### 2.2. Design of Functionally Graded Scaffolds Based on TPMS

Design of personalized scaffolds to replace damaged bone tissue requires consideration of many different factors, such as additive manufacturing parameters [26], as well as careful selection of the basic geometry that will form the basis of the scaffold [27]. In this study, triply periodic minimal surfaces (TPMS) were used as the basic unit cell due to their advantages in terms of a number of mechanobiological parameters, as well as the possibility to control the morphology of the structure [28,29].

TPMS divide the volume, which is considered as a unit cell, into two different sub-areas [30]. In general, the TPMS function is defined by the following expression:

(1) $$\phi (r)=\sum_{l=1}^{L}\sum_{m=q}^{M}\mu_{ml}\cos(2\pi \kappa_{l}(P_{m}^{T}r))+Q=0,$$

where $\mu_{ml}$ is a periodic moment, $\kappa_{l}$ is a scale factor, $P_{m}=\left(a_{m},b_{m},c_{m}\right)$ is a basis vector, $r=\left(x,y,z\right)$ is a local vector, *Q* is a parameter that controls the total porosity of the structure. The gradient of morphology allows pores shape in the structure to change continuously by joining together different TPMS-based unit cells. To obtain a gradient transition from one cell morphology to another, the structure can be given by the following expression [31]:

(2) $$\phi_{M}\left(r\right)=\sum_{i=1}^{m}\frac{\phi_{i}\left(r\right)}{e^{k{\left\|r-r_{i}\right\|}^{2}}}\leq 0,$$

where $\phi_{i}\left(r\right)$ are the expressions for selected types of TPMS, $e^{-k{\left\|r-r_{i}\right\|}^{2}}$ are weighting coefficients, *k* is a transition parameter between TPMS cells, the increase in which leads to a sharper transition between morphologies, $r_{i}$ are the coordinates of points were transition from one type of morphology to another occurs, *m* is a number of substructures with different morphologies united within one structure. In this study, the gradient transition is implemented between two types of TPMS cells along the Y-axis.

In this paper we consider functionally graded structures that are formed on the basis of two types of TPMS: diamond and I-WP. For the convenience of notations, the following designations were introduced: D is a diamond surface and P_II_ is a I-WP-surface (Table 3).

Here, $\alpha =\beta =\gamma =\frac{2\pi n}{\left(x_{\max}-x_{\min}\right)}$ is a scaling factor, *n* is a number of unit cells along the X axis. In this case study, a single cell along the X axis was used for both surface types. The space occupied by each designed structure is bounded by the region Ω:

$$\Omega =\left\{\left(x,y,z\right)\phi \left(x,y,z\right)\leq 0,x_{\min}\leq x\leq x_{\max},y_{\min}\leq y\leq y_{\max},z_{\min}\leq z\leq z_{\max}\right\}$$

where $\left(x,y,z\right)$ is a global coordinates of the area, $\phi \left(x,y,z\right)$ is a surface based on TPMS, $x_{\min}$=$y_{\min}$=$z_{\min}$= 0, $x_{\max}$=$z_{\max}$= 5, $y_{\max}$= 15 are the structure boundaries in millimeters.

**Figure 2:**
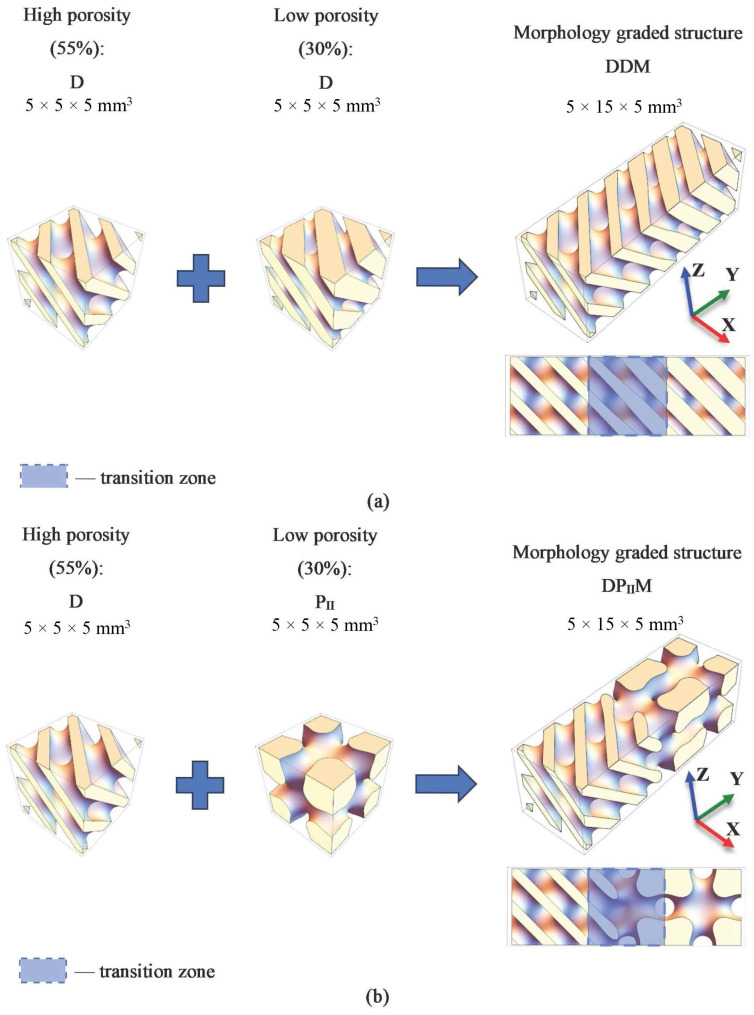
Visualization of structures with morphology gradient: (**a**) DDM; (**b**) DP_II_M.

Two types of structures with the gradient of morphology were designed. The architecture of each structure consisted of three equal parts: a highly porous part that mimics trabecular bone tissue, a smooth gradient transition between the morphologies, and a part with a low pore volume fraction that mimics cortical bone tissue (Figure 2). For simplicity, the following structure designations are introduced: DDM (high-porosity part is based on D surface and low-porosity part is based on D surface) and DP_II_M (high-porosity part based on D surface and low-porosity part based on P_II_ surface).

These models were used for manufacturing of PLA specimens that were investigated in a series of mechanical compression tests before and after the degradation process.

### 2.3. 3D Printing and Mechanical Testing

**Figure 3:**
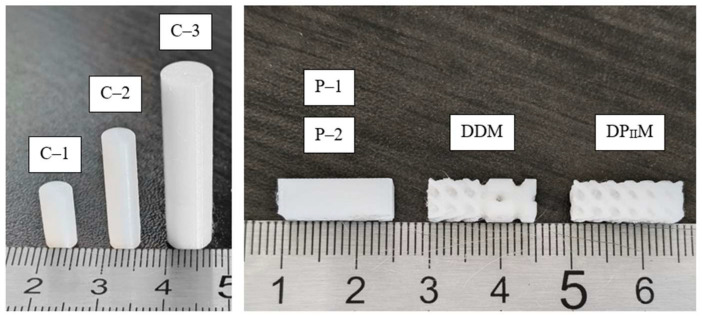
Images of samples produced from PLA.

Solid cylindrical and prismatic samples, as well as porous samples were manufactured from PLA using fused filament fabrication (FFF) technology (Figure 3). A Raise3D Pro3 3D printer (Raise3D, Irvine, CA, USA) was used with the following printing parameters: nozzle diameter of 0.2 mm, print speed of 300 mm/min, layer height of 0.1 mm, bed temperature of 65 °C, and nozzle temperature of 225 °C. The samples were tested under uniaxial compression on an Instron 68SC-5 testing machine (Norwood, MA, USA) with a 5 kN load cell and an AVE2 video extensometer. The tests were conducted at room temperature. Lubrication layers and Teflon sheets were placed between the plates and the sample to minimize friction during testing. A high-temperature lubricant, Cyatim-221F, with ultradispersed polytetrafluoroethylene (PTFE), was used. A PTFE film with 50 µm thickness significantly reduced surface friction. To remove excess lubricant before testing, a cycle with preloading up to 5 N and unloading down to 0 N was performed in five iterations. This procedure allowed for levelling the lubricant layer. The load was applied both along the direction of printing (i.e., the layers were perpendicular to the load for solid cylindrical and prismatic samples) and at 90° relative to printing (i.e., the layers were aligned along the load direction for solid prismatic and porous gradient samples), with a loading speed of 1 mm/min.

The effective elastic response (modulus of elasticity) and compressive strength were determined for all specimens based on the results of the loading. The ultimate strength can be determined from the maximal stresses values occurring during loading of the specimen. There is also an approach to determine the ultimate strength based on the dependence of the instantaneous modulus of elasticity on strain [32]. In this case, the inflection point on the graph of the dependence “instantaneous modulus of elasticity—strain” is characterized by the ultimate strength. The discrepancy between the results obtained using these two approaches did not exceed 1% (Appendix A). Therefore, within the framework of this work, the ultimate strength was determined by the maximum stresses.

### 2.4. Conditions of Degradation

The influence of the liquid medium on the mechanical properties of the specimens was investigated. Two degradation modes were proposed (Table 4). In the D–1 mode, the specimens interact with the physiological solution at a human body temperature (37 °C). Mode D–2 (elevated temperature of 45 °C) was proposed to evaluate the effect of temperature factor on the degradation rate of elastic properties. The influence of the proposed degradation modes on the mechanical properties of the samples was investigated after three, seven, eleven and fourteen days of exposure.

The samples in a closed container were placed into a Binder FP53 temperature chamber (BINDER GmbH, Tuttlingen, Germany). After reaching the required number of days, the samples were removed from the container and subjected to mechanical testing; before testing, the samples were cured until the surface was dry.

### 2.5. Microcomputed Tomography

Microcomputed tomography (micro-CT) was employed to assess printing quality, to verify the compliance of the obtained samples’ architecture with the original models, and to identify structural changes that may influence the mechanical properties of the structures during degradation. An Xradia Versa 520 device (Carl Zeiss Xray Microscopy, Inc., Pleasanton, CA, USA) with the following identical parameters for each sample was used: 0.4X objective (with a pre-installed scintillator), tube voltage of 80 kV, power of 6.5 W, pixel size of 21.5 µm, sample rotation of 360°, exposure time of 1 s, and no filter on the X-ray tube. During microtomography, 1601 projections of each sample were obtained. The reconstruction of the projection set into a series of virtual sections was performed using XRMReconstructor software version 12.0., with center offset values determined manually. A Gaussian blur filter (0.5) was applied, and the beam spectrum was shifted to a harder (high-energy) region (standard value 0.05). To correct sample drift during scanning, an option for additional compensatory movements was used. A filter on the X-ray tube LE2 was used to correct ring artifacts.

Before each scan, a warm-up scan was performed. For the post-processing of the virtual slice sets of the samples, VGSTUDIO MAX 3.5 software (Volume Graphics GmbH, Heidelberg, Germany) was used, resulting in three-dimensional virtual models of each sample. For three-dimensional visualization, rendering was performed using the Phong shading model [33].

### 2.6. Morphometric Analysis

Morphometric analysis was used for comparing the data on the internal architecture of samples obtained by micro-CT with the data on the microstructure of numerical models. The parameters adopted in tissue engineering to describe the structure of trabecular bone tissue were used as metrics: the average volume fraction of pores in the sample (BV/TV), the internal surface area of the structure (Tb.BS, mm^2^), the average thickness of structural elements (Tb.Th, mm, for bone—the average thickness of trabeculae) [10,34,35,36,37,38,39,40].

To calculate the average density/porosity in the case of TPMP-based structures, analytical methods for volume calculation are applicable (3):

(3) $$\rho =\frac{{∭}_{\Omega }dv}{\left(x_{\max}-x_{\min}\right)\left(y_{\max}-y_{\min}\right)\left(z_{\max}-z_{\min}\right)}=1-p,$$

$$\Omega =\left\{\left(x,y,z\right)\phi \left(x,y,z\right)\leq 0,x_{\min}\leq x\leq x_{\max},y_{\min}\leq y\leq y_{\max},z_{\min}\leq z\leq z_{\max}\right\}.$$

where *ρ* is relative density, *p* is porosity, and the indices max and min are spatial dimensions of the structure.

To determine the volumetric local thickness τ of the structure, the diameter of the largest of the spheres inscribed in the structure containing the local point was taken. This technique was proposed as a solution to the problem of accurate description of the morphometry of complex spatial structures [41]. The average thickness of the scaffold was defined as an arithmetic mean of the local thickness taken over all points of the structure. Similar calculations are applicable for estimation of pores diameters. For the obtained data, the standard deviation of the mean thickness value was calculated, which can be used for more accurate separation or classification of different structures.

The area of the inner surface was determined by the surface integral of the first kind from the unit function over the surface of the structure (4):

(4) $$S_{\phi }=\iint_{\phi }d\phi \left(x,y,z\right)$$

To calculate the specific surface area (SSA, mm^−1^), the internal surface area was normalized to the volume of the structure. In the presented study, based on the results of micro-CT, the volume and specific surface area of the scaffold prototypes under consideration were calculated using VGstudio Max 3.5 software, while the average wall thickness of the structure was determined using the ImageJ software v1.53t (BoneJ plugin).

## 3. Results

### 3.1. The Effect of the Degradation Mode on the Mechanical Properties of Solid Specimens

**Figure 4:**
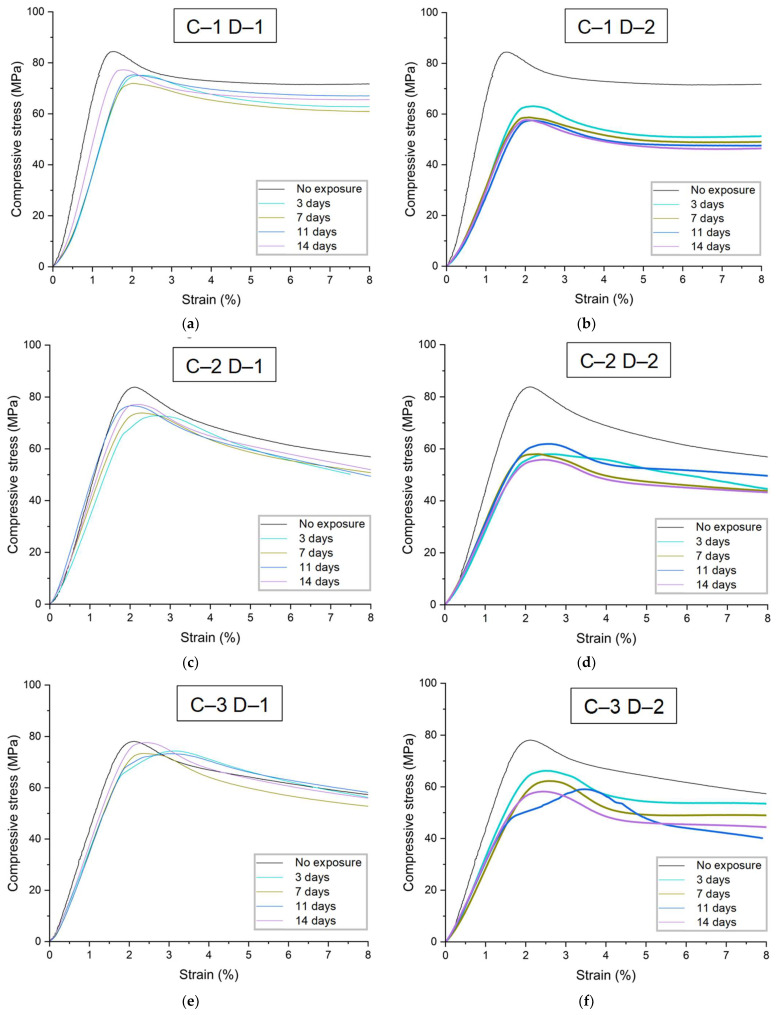
Averaged stress–strain curves for continuous cylindrical specimens: (**a**) specimens C–1 under degradation regime D–1; (**b**) specimens C–1 under degradation regime D–2; (**c**) specimens C–2 under degradation regime D–1; (**d**) specimens C–2 samples under degradation regime D–2; (**e**) C–3 samples under degradation regime D–1; (**f**) C–3 samples under degradation regime D–2. The data with the confidence interval for all presented experimental curves are presented in Appendix B.

Uniaxial compression tests were performed on cylindrical specimens to evaluate the influence of environmental parameters on the mechanical properties of the PLA material. Experimental stress–strain dependences were obtained from the results of compressive tests (Figure 4). A series of five specimens for each type of tests were studied during different exposure periods (no exposure, 3, 7, 11 and 14 days of exposure) at different temperature regimes (D–1 and D–2, respectively). The results for samples without degradation were chosen as a reference for comparison.

According to the data obtained, exposure to degradation mode D–1 (37 °C, Figure 4a,c,e) affects the mechanical properties of the material to a much lesser extent than degradation mode D–2 (45 °C, Figure 4b,d,f). Changes were observed both in the elastic properties and in the mechanical behavior—for example, the compressive strength of the material decreases. At the same time, the rate of degradation of properties and the nature of changes were different for each group of specimens. Group C–1 was characterized by a 30% decrease in the elastic modulus on day 3 in degradation mode D–1 relative to reference data, and by 40% in mode D–2. For group C–2, there was a 22% decrease in the elastic modulus in the D–1 degradation mode and a 35% decrease in the D–2 degradation mode over the same time period. For group C–3, the elastic modulus decreased by 16% and 22% for degradation modes D–1 and D–2, respectively, over the same time period. Thus, the degradation rate directly correlates with both ambient temperature and sample size.

**Figure 5:**
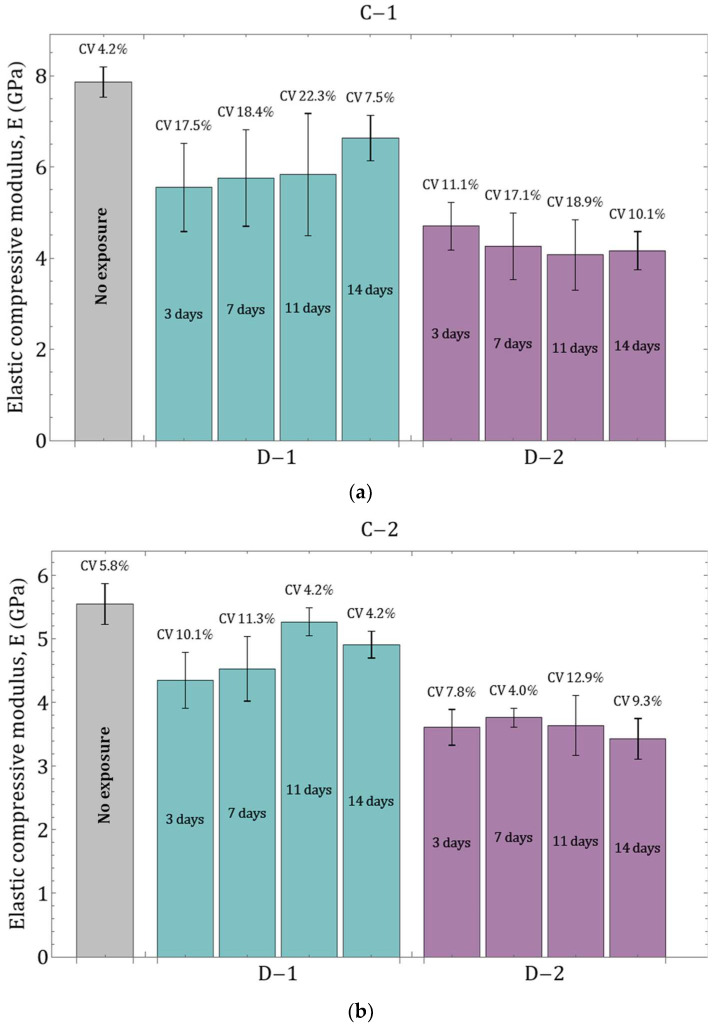

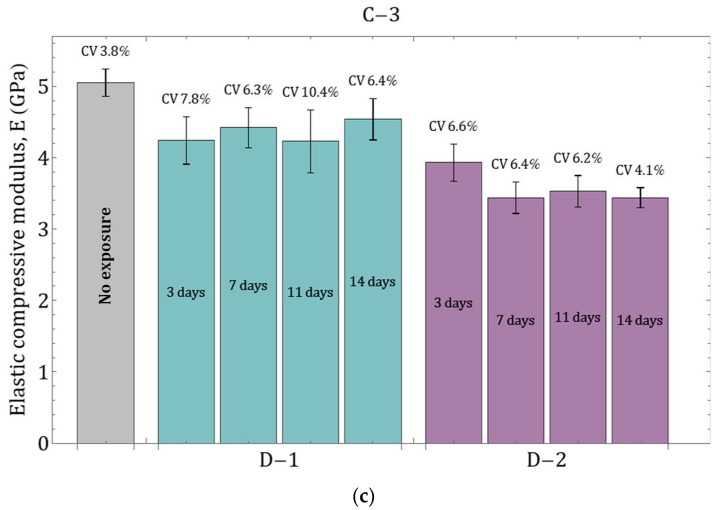
Variation in the compressive elastic modulus of cylindrical specimens depending on the degradation mode: (**a**) specimens C–1; (**b**) specimens C–2; (**c**) specimens C–3.

During degradation in mode D–1 after the first stage (3 days), a gradual increase in the elastic modulus was observed for samples C–1 (19.5% increase on 14th day), an increase, followed by a decrease, for samples C–2 (21.1% increase on 11th day, 7% decrease on 14th day), and minor fluctuations for samples C–3 (7% increase on 14th day) (Figure 5). This behavior of the material may be related to the internal architecture of the samples manufactured using additive technologies: as the samples in the liquid medium gradually become “saturated” (the liquid penetrates the space between the filaments), they are subjected to testing as moisture-saturated structures. In this case, three factors influenced mechanical properties: temperature, exposure to the liquid medium, and the degree of moisture saturation. Depending on the size of the samples (C–1, C–2, and C–3), complete filling of the inter-filament space will require more time; thus, structures with fully liquid-filled pores will demonstrate stiffer behavior in the early stages of degradation. Therefore, the increase in elastic moduli observed for structures C–1 during degradation mode D–1 may be attributed to a low rate of material degradation at the given temperature regime and simultaneously high moisture saturation. For samples C–2, the observed increase in properties may be associated with the same factors, while differences from results for samples C–1 can be attributed to size differences and, consequently, a longer period for complete “saturation” of the sample. For samples C–3, the observed deviations may be related to both the factors mentioned above and measurement errors, since the obtained average values differ only slightly (coefficient of variation, CV, was 3.4%). On the other hand, unstable changes in elastic properties over time may be linked to a larger confidence interval at specific stages of degradation research: the standard deviation from the mean for samples C–1 ranged from 7.5% to 22.3% (average 16.4%), for samples C–2 from 4.2% to 11.3% (average 7.5%), and for samples C–3 from 6.3% to 10.4% (average 7.7%). For degradation mode D–2, from days 3 to 14, a slow decrease in the elastic modulus was characteristic; here, the CV for samples C–1 fluctuated from 10.1% to 18.9%, averaging 14.3%, for samples C–2 from 4% to 12.8% (average 8.5%), and for samples C–3 from 4.1% to 6.4% (average 5.8%). Overall, the accuracy of determining the elastic modulus under compression was higher for samples C–2 and C–3, while for samples C–1, the spread of obtained values was significantly greater.

In addition to the elastic modulus, data on the compressive strength values for all tested samples were obtained (Table 5). This parameter was determined with good accuracy: for the series of tests on samples without degradation, the coefficient of variation (CV) did not exceed 3%, while for different stages of degradation, the CV ranged from 1% to 11.9%, averaging no more than 6.7%. The compressive strength of samples C–1 in degradation mode D–1 decreased by 10.5% on the third day, with discrepancies from data obtained at subsequent stages of degradation not exceeding 3%. For C–2 samples, the decrease in compressive strength on the third day of degradation in mode D–1 was 9.2%, with changes at subsequent stages not exceeding 3%. In the case of C–3 samples, the compressive strength decreased by 0.7% on the third day of degradation, with changes at subsequent stages not exceeding 3.5%. Thus, unlike the results obtained for the elastic modulus under compression, degradation mode D–1 had a significantly lesser effect on the strength properties of the samples. For samples C–3, the observed decrease in strength properties during the observed time period was minimal and may be associated with measurement errors. In degradation mode D–2, the decrease in strength properties on the third day was 27.2% for samples C–1 and C–2 and 13.2% for samples C–3. At subsequent time periods, properties continued to decline—by the fourteenth day, compressive strength decreased relative to the initial material properties by 31.0%, 32%, and 24.7% for samples C–1, C–2, and C–3, respectively.

It is worth noting that the initial elastic moduli of the material (Table 5) differed for samples C–2 and C–3 within the measurement error range, while the calculated elastic modulus for structures C–1 was significantly higher. Additionally, samples C–1 generally exhibited a greater spread of measured values (see Figure 5, Table 5). The instability of the obtained results may be related not only to the effects of various degradation modes but also to the small dimensions of the samples and the limitations of FFF printing technology [32]. Furthermore, the data obtained for the elastic modulus may vary because the objectives of this study did not allow for the use of samples the dimensions of which would comply with ASTM D695, which is designed to evaluate the properties of plastics under compression. Since the necessary ratio between the diameter and height of the sample for correct determination of the elastic modulus was not met, the results obtained for each type of sample represented an effective response of the structure to the applied load.

### 3.2. Impact of Printing Orientation on Mechanical and Degradation Properties

**Figure 6:**
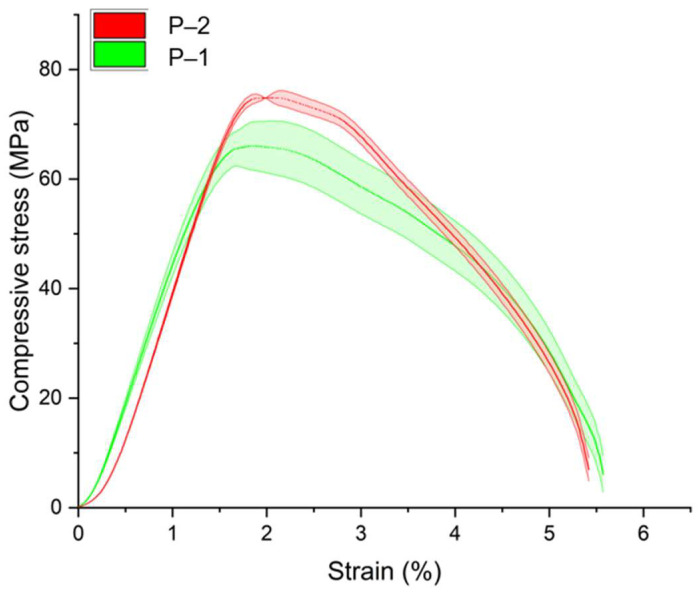
The effective response of samples with different layer orientation under compressive loading.

The effect of the orientation of the layers relative to the applied load on the mechanical and degradation behavior of structures was evaluated. To evaluate the effect of printing orientation on mechanical properties, compression tests were performed over solid prismatic images (Figure 6). The specimens were printed in two orientations: vertical (P–1) and horizontal (P–2).

The test results for specimens P–1 and P–2 showed an insignificant change in the elastic modulus depending on the printing orientation. The same was observed for the obtained averaged curves. At the same time, a greater scatter of experimental curves in the zone of nonlinear behavior was observed for P–1 specimens. The elastic and strength properties in compression in the case of vertical printing orientation decreased relative to horizontal printing orientation for both solid and lattice specimens (Table 6).

The effective elastic response to compression for solid samples P–1 and P–2 differed by 4.9%. For samples with a horizontal printing orientation, the discrepancy between the obtained parameters was less than for samples with a vertical orientation: the coefficient of variation (CV) for the effective elastic response to compression for samples P–1 is 3.6%, while for samples P–2, the variation was 0.5%. For samples P–1, a decrease in compressive strength of 10.8% relative to samples P–2 was observed, with CV values of 5.7% and 0.9% for P–1 and P–2, respectively. Overall, the printing orientation has a negligible effect on the compressive mechanical properties of the structures. However, orientation can impact the print quality of scaffolds with complex architectures. The results obtained for samples manufactured in a vertical orientation showed greater variability in elastic response compared to those made in a horizontal orientation.

**Figure 7:**
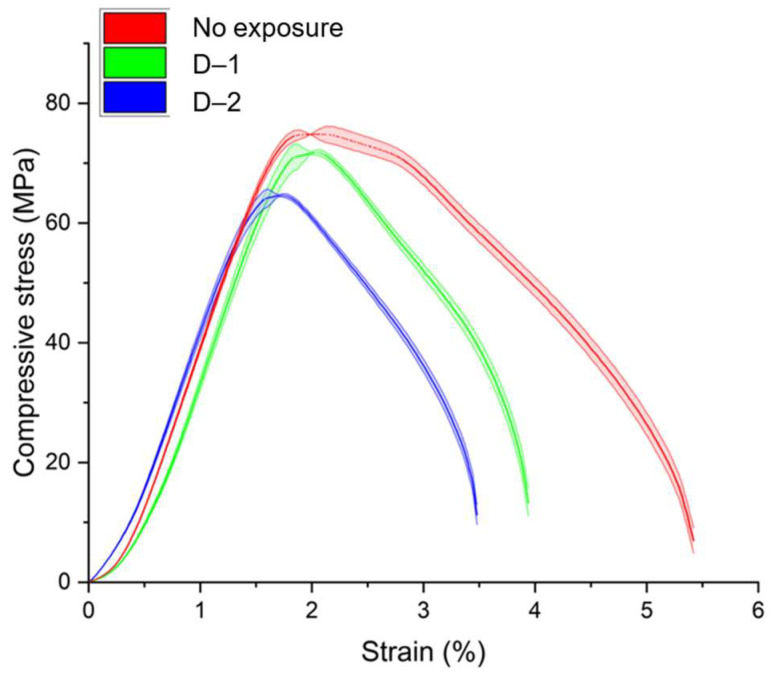
The effect of degradation modes on the mechanical behavior in compression for continuous prismatic specimens P–2.

To assess the impact of printing orientation on the degradation rate during immersion in a liquid medium, P–2 samples were subjected to degradation in modes D–1 and D–2. The results obtained for the samples exposed for two weeks were compared with the reference values obtained for samples without degradation (Figure 7).

The obtained results are in good agreement with those previously obtained for solid cylindrical samples: degradation mode D–1 had a significantly lesser impact on mechanical properties compared to degradation mode D–2. The reduction in the effective elastic modulus under compression relative to reference data was 0.5% in degradation mode D–1 and 3.2% in degradation mode D–2. For the strength limit, the reductions were 3.5% and 13.3%, respectively. There was also a slight discrepancy observed both between the experimental stress–strain curves and for the calculated mechanical characteristics (Table 7).

The effective elastic compressive response and ultimate strength were determined with high accuracy both for specimens without degradation and for specimens subjected to different degradation regimes. The relative standard deviation did not exceed 2%, which indicates the reliability of the results obtained.

### 3.3. The Effect of the Degradation Mode on the Mechanical Characteristics of TPMS Structures

**Figure 8:**
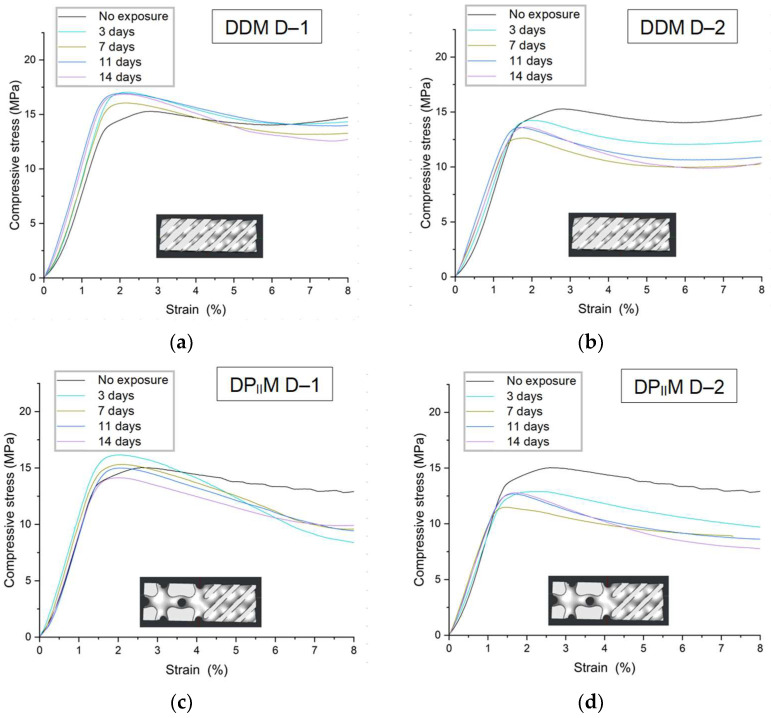
Averaged experimental stress–strain curves for lattice specimens: (**a**) DDM, D–1 degradation mode; (**b**) DDM, D–2 degradation mode; (**c**) DP_II_M, D–1 degradation mode; (**d**) DP_II_M D–2 degradation mode. The data with the confidence interval for all presented experimental curves are presented in Appendix B.

In order to evaluate the effect of degradation on the mechanical properties of TPMS-based scaffolds, mechanical compression tests were performed on DDM and DP_II_M lattice specimens fabricated in horizontal orientation and subjected to degradation modes D–1 and D–2. Similar to the tests performed for solid specimens, the mechanical behavior of each type of structures was evaluated for each degradation mode at several time periods (Figure 8). The averaged test results of structures without exposure were taken as a reference.

During the examined time interval, degradation mode D–1 did not affect the elastic properties of the structure (see Appendix C). The change in effective elastic response for DDM structures did not exceed 4.1%, with parameter determination errors ranging from 1.6% to 7.9%. For DP_II_M structures, the deviation from the reference value did not exceed 3.3%, and the error was no more than 7.6%. In degradation mode D–2, there was a reduction in elastic response of 15.6% for DDM structures and 10% for DP_II_M structures, with parameter determination errors fluctuating between 6.5% and 14.5% for DDM and between 5.8% and 16% for DP_II_M. In both cases, the rate of degradation was quite low: in mode D–1, the influence was negligible, while in mode D–2, after an initial sharp change in properties (on day 3), further influence on elastic properties was minimal.

The obtained strength limit values were more sensitive to environmental influences compared to the elastic response (Table 8). In degradation mode D–1 for the DDM structure, the strength limit increased by 9.3% on the third day and gradually decreased in subsequent stages. A sharp decline in modulus was observed on the seventh day, which may be associated with molecular changes in the structure during degradation or with printing defects. A similar behavior was observed for the DP_II_M structure: at the initial stage of degradation, there was an increase in strength limit (by 4.7%), followed by a gradual decrease at later stages. The average strength limit on the 14th day of degradation was 8.2% lower than the value for samples without exposure.

In degradation mode D–2, a significant decrease in the structural strength values was observed. At the initial stage (3 days), the degradation of DDM structures was 7.4%, which increased to 12.6% by the end of the observed time period. For DP_II_M structures, the initial decrease in strength properties was 14%, which further increased down to 17.1%. In general, it can be stated that the strength properties of the DP_II_M structure degraded faster than those of the DDM structure. This may be related to the differences in morphometric parameters as well as to the manufacturing-induced defects, which could appear because of the complex architecture of the samples.

### 3.4. Investigation of Internal Morphology and Defects

**Figure 9:**
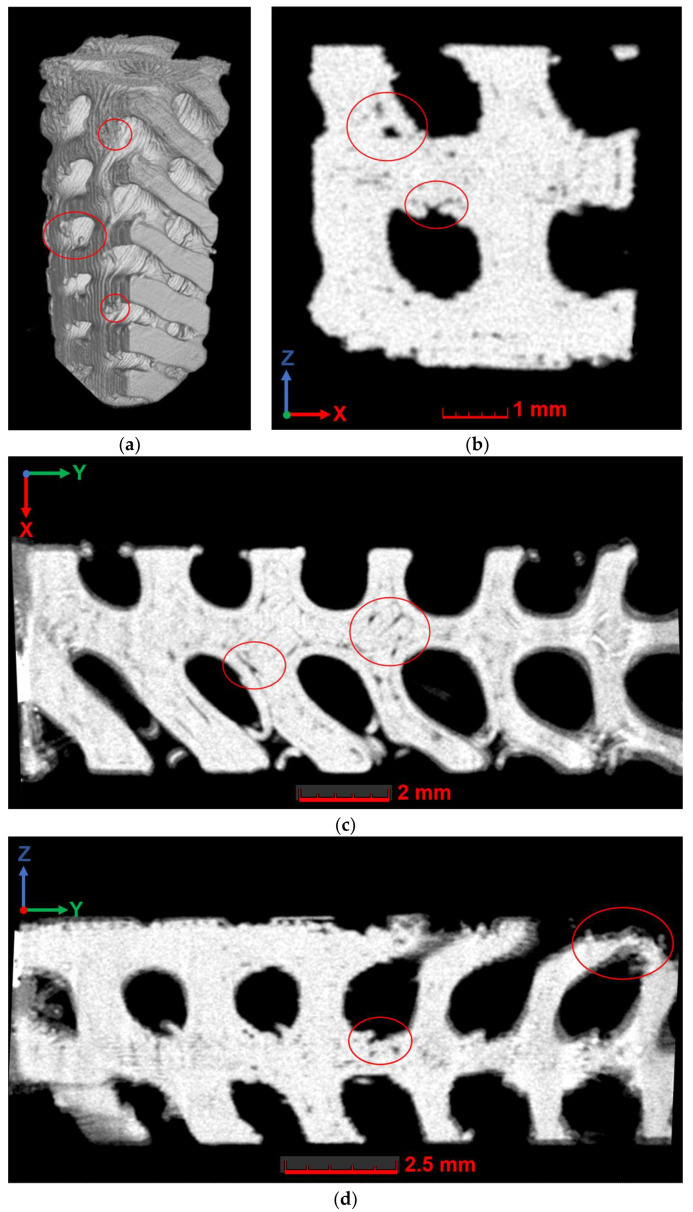
The results of microtomography for the DDM sample without degradation: (**a**) three-dimensional representation; (**b**) cross-section in the XZ plane; (**c**) cross-section in the XY plane; (**d**) cross-section in the YZ plane. Defects are marked with red circles.

**Figure 10:**
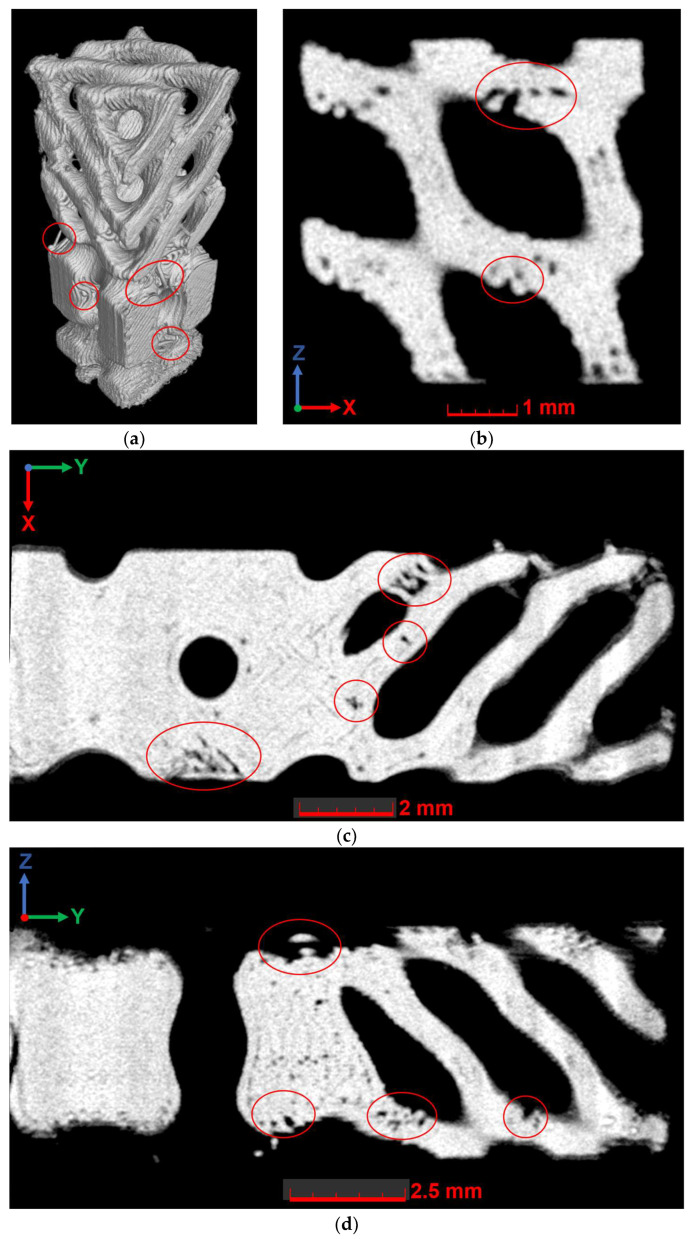
The results of microtomography for the DP_II_M sample without degradation: (**a**) three-dimensional representation; (**b**) cross-section in the XZ plane; (**c**) cross-section in the XY plane; (**d**) cross-section in the YZ plane. Defects are marked with red circles.

Tomography studies were conducted to evaluate the possible influence of the degradation process on the internal architecture of fabricated lattice specimens. The analysis of the internal architecture of the samples was conducted both for volumetric reconstructions of the structure and individual cross-sections (see Figure 9 and Figure 10). Material boundaries were identified based on local variations in grayscale values (ranging from 0 to 65,535 arbitrary units). Compared to the determination of the sample material’s surface based on global features (using one single grayscale value applied globally to the dataset), enhanced definition enabled more precise segmentation of geometric structures, as local deviations caused by other factors (e.g., artifacts) were largely compensated.

In the examined structures, voids were locally found in the interlayer space, individual voids within the polymer layer (filling defects), as well as accumulations of polymer outside the designated structure. Filling defects were associated with the complex architecture of the sample, as the nozzle could not fill areas with a smaller diameter than that of the nozzle itself. Furthermore, the complex architecture of the sample does not allow for the use of support structures during printing because they cannot be removed from the internal part of the structure. Consequently, some of the observed defects were related to the fact that individual elements of the structure were printed at a large overhang angle.

**Figure 11:**
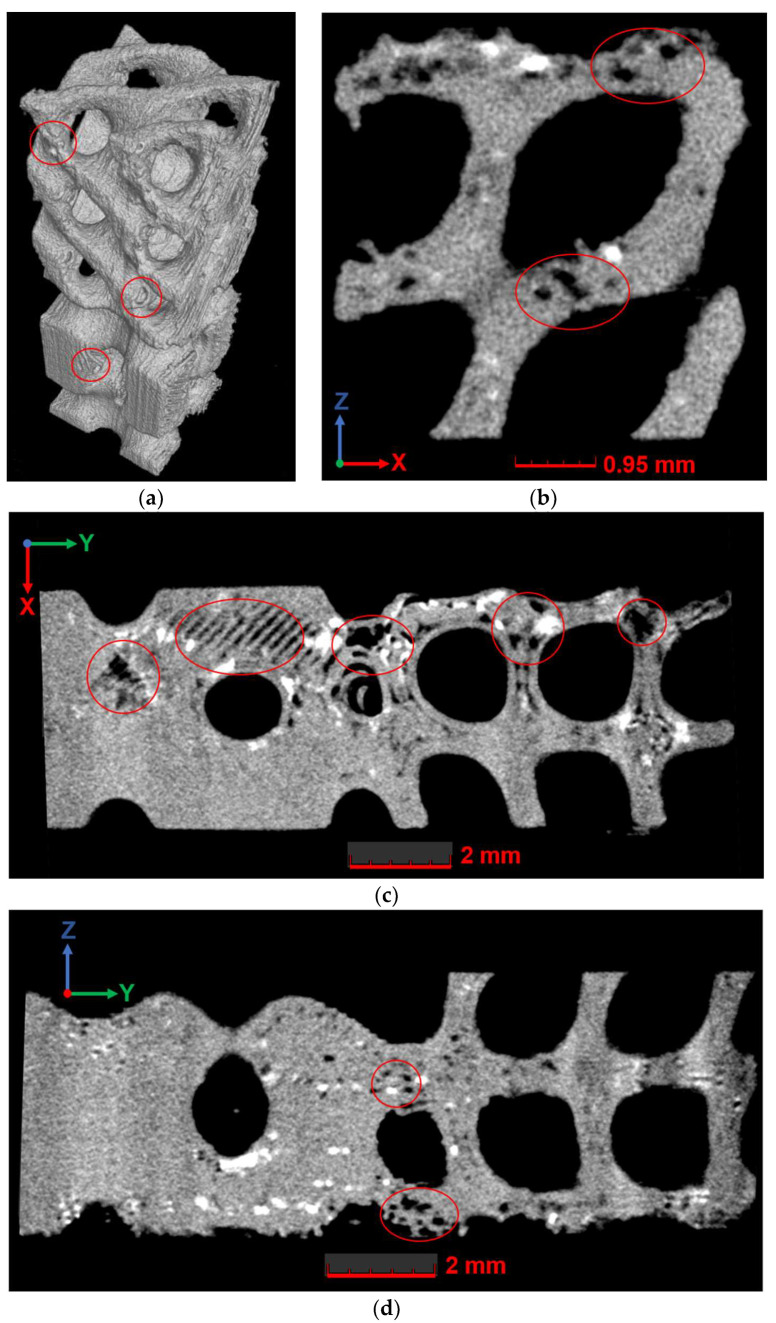
The results of micro-CT for the DP_II_M sample with degradation in mode D–1 on day 14: (**a**) three-dimensional representation; (**b**) cross-section in the XZ plane; (**c**) cross-section in the XY plane; (**d**) cross-section in the YZ plane. Defects are marked with red circles.

There was a multiple increase in internal defects in structures that have undergone degradation. For instance, in the DP_II_M structure, which was immersed in a liquid medium at 37 °C for 14 days (degradation mode D–1), analysis of individual slices revealed significantly more number internal defects than in control samples with no exposure (Figure 11).

The location of the observed defects in the slices of the structure exposed to the liquid corresponded to the location of previously observed manufacturing-induced defects in the control samples. Locally, larger individual defects (Figure 11c) were observed compared to those previously found in the control group samples with no exposure. For another type of structure and for other degradation modes, the observed pattern corresponds to the discussed one (see Appendix D for more details).

To assess the change in the internal structure of scaffolds, a morphometric analysis was conducted. Using the Advanced classic algorithm [42], with automated contour searching in the vicinity of the densest areas of the internal structure of the sample and a search distance of 0.861 mm, the surface of the scaffold material was determined. Based on these data, the volume and specific surface area of the samples were calculated, and the average wall thickness of the samples was determined using the BoneJ plugin (Table 9).

As the liquid medium exerts its influence, a gradual decrease in the volumetric fraction of the remaining structural material was observed for the studied samples. Additionally, there was an increase in the specific surface area of the samples, indicating surface erosion. At the same time, the calculated average wall thickness of the structure also showed a tendency to thinning compared to the control group of samples.

## 4. Discussion

Experimental studies on the change in the mechanical response of solid samples subjected to the influence of a liquid medium and temperature showed the following: on the third day of exposure, a decrease in elastic moduli was observed compared to the control group of samples. Depending on the types of samples and the degradation mode applied, the reduction in the elastic modulus ranged from 16% to 40%. In the subsequent days, a gradual increase in the elastic modulus was observed, which was apparently related to the polymer absorbing the liquid, as well as the liquid seeping into the spaces of manufacturing-induced porosity within the sample. As a result, the effective properties obtained correspond to a moisture-saturated material in which the process of degradation of mechanical properties has begun [18]. In [43], a similar study was carried out for highly porous (60% and 80%) mesh scaffolds for 50 days. A significant effect of pore size on surface erosion and mass loss of the scaffolds was found, and a significant reduction in the mechanical properties was also recorded.

Printing orientation, as was shown, had no effect on mechanical response of the studied prototypes; therefore, the choice of printing direction should be determined solely by technical limitations associated with the complex architecture of the fabricated sample [44].

**Figure 12:**
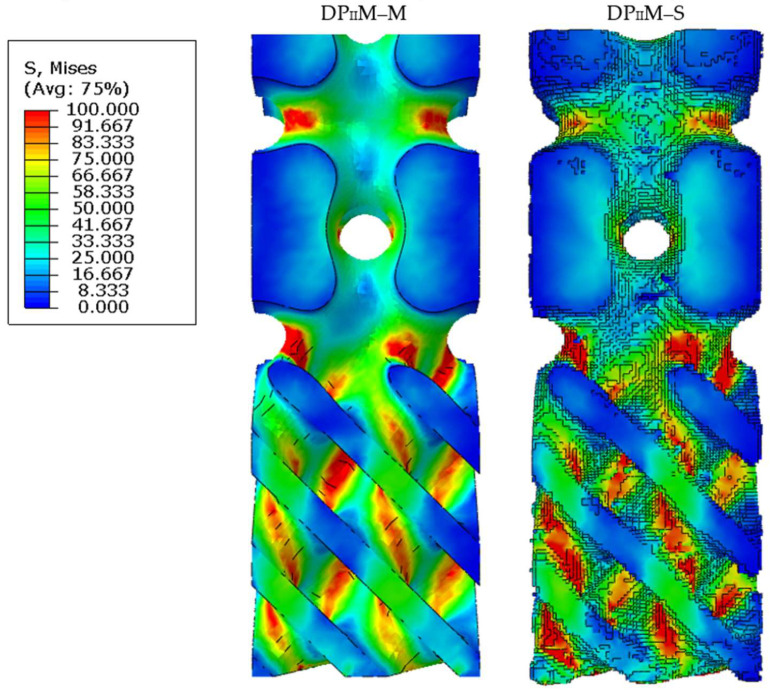
Equivalent Mises stress distributions over the volume of the initial structure (DP_II_M–M) and voxel-based reconstruction of the real sample (DP_II_M–S).

Since the structure of the real sample always differs from its numerical prototype, the differences in their mechanical response were of interest. For this purpose, based on the results of micro-CT, a model of an actual DP_II_M structure (DP_II_M–S) was reconstructed using a program code developed in the Wolfram language within the Mathematica computer algebra system. The reconstruction was performed for a sample without exposure from the control group. The designed model of the DP_II_M structure (DP_II_M–M) was used for comparison. Finite element analogs of both models were created, and numerical calculations for axial compression were performed using the SIMULIA Abaqus application package. The calculation was performed in a linear-elastic formulation. A displacement of 0.15 mm (corresponding to 1% macro-deformation of the structure) was applied to the upper face of each structure. The material properties were defined according to those determined for solid prismatic samples P–2: elastic modulus E = 5550 MPa, strength limit = 72.5 MPa, and Poisson’s ratio = 0.36 [45]. Based on the calculation results, a qualitative assessment of the distribution of equivalent stresses (according to von Mises) in the structure was conducted (Figure 12).

**Figure 13:**
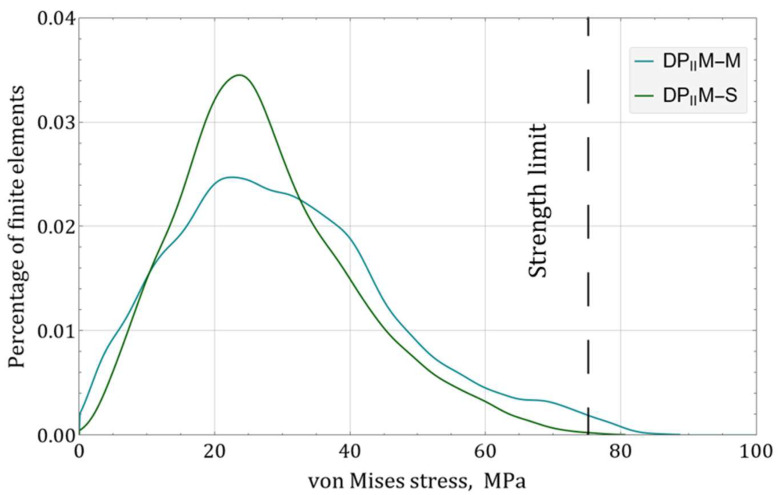
Distribution of equivalent stresses (by Mises) over the volume of the structure.

For the models under consideration, there was a good qualitative similarity in terms of the location of potential stress concentrators within the structure, as well as in terms of the stress values at these locations. However, in the DP_II_M–S, the highly porous lower part of the construction was more distorted compared to the original. To evaluate how the observed deviations from the original geometry affect the mechanical response of the structure, distributions of equivalent von Mises stresses were constructed for the studied structures based on statistical analysis methods for random variable distributions [46] (Figure 13).

According to the obtained results, the observed distortions in the geometry of the sample relative to the original model had a noticeable impact on the stress distributions. The original model demonstrates more ductile behavior than the model derived from the computer tomography results. Hence, it can be concluded that the original numerical model predicts the mechanical response of scaffold prototypes with fairly good accuracy; however, real samples possess a greater safety margin, according to the right part of the distributions. Thus, this feature can be taken into account during the design phase by introducing some safety factor into the numerical model, or the original numerical model can be refined based on the results of computer tomography. It is worth noting that the identified discrepancies between the models were not critical.

The changes in the structure of the samples identified during microtomography and morphometric analysis allow to draw the following conclusion: the liquid medium and exposure to elevated temperatures have a significant impact on the surface condition of the structure, even in the absence of the fluid flow. In the case of dynamic fluid flow through, the process of surface erosion is likely to be accelerated. The fact that the extent of liquid absorption is highly influenced by the structure of the scaffold is in correspondence with the other published results. In [47], the degradation studies were performed for the scaffolds with triangular, rectilinear and honeycomb infill patterns. A gradual decrease in strength properties was shown, as well as the influence of the internal architecture on the degradation rate. In [48], the degradation rate of scaffolds with cubic and body centered cubic structure was evaluated within 120 days. It was shown that in PLA scaffolds can continuously absorb liquid for a long period of time (up to 60 days), what directly affects the change in their mechanical properties. Although the general tendency of change in mechanical properties is observed, the occurrence of surface erosion and the relationship of these phenomena with the morphometric parameters of the structure cannot be generalized to arbitrary geometry of the scaffold. The presented study extends the understanding of these processes for the case of solid specimens and functionally graded TPMS-based structures.

It should be noted that this study only considered the period of interaction between polymer samples and the liquid corresponding to a two-week duration. For a more complete understanding of the degradation mechanism of such objects, it is necessary to examine later stages of degradation and how the properties of the samples will change in that case. Additionally, since the samples were tested in a moisture-saturated state, this work did not precisely determine how the material properties changed relative to the initial state. Future studies should include testing of dried samples after exposure to the liquid medium and various temperatures to accurately assess the rate of degradation of the material properties.

## 5. Conclusions

The present study reports an experimental assessment of the changes in the mechanical properties of polymer structures immersed into a liquid medium under various temperatures over different periods. An increase both in the elastic modulus and compressive strength was observed for solid samples at the initial stages of degradation associated with the gradual saturation of the polymer with liquid. After this, the mechanical properties of moisture-saturated solid samples began to decrease. During this period, the effective elastic modulus decreased more rapidly in degradation mode D–2 (approximately 40%), while in mode D–1, the reduction was insignificant (approximately 12%). The results of mechanical tests on prototypes of lattice scaffolds aligned well with those for solid samples: they also exhibit an increase in both strength and elastic properties at the initial stage of interaction with a liquid environment and sensitivity to temperature effects. As a result of morphometric analysis of the stack of 2D slices of samples obtained using micro-CT, the beginning of surface erosion in lattice samples was detected even at the early stages of degradation. Numerical analysis of the stress distribution in the sample reconstructed based on tomography results showed that the real structure resisted compressive loads better than the model. The obtained results expand the understanding of the degradation process of polymer structures intended for biomedical applications and may be useful in modelling of the interaction of scaffold prototypes with biological environments to predict their operational characteristics.

## Figures and Tables

**Table 1:** The geometric characteristics of cylindrical solid specimens.

Sample’s Type	Diameter, mm	Height, mm	Printing Orientation	Visualization
C–1	5	10	vertical	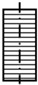
C–2	5	20		
C–3	7.5	30		

**Table 2:** Geometric dimensions and printing orientation of prismatic specimens.

Sample’s Type	Section Dimensions, mm	Height, mm	Printing Orientation	Visualization
P–1	5 × 5	15	vertical	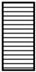
P–2	5 × 5	15	horizontal	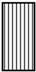

**Table 3:** Visualization and analytical expression for TPMSs.

Structure	TPMS Expression
D 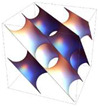	$\sin\left(\alpha x\right)\left(\sin\left(\gamma y\right)\sin\left(\beta z\right)+\cos\left(\gamma y\right)\cos\left(\beta z\right)\right)+\;\;\;\;\;+\cos\left(\alpha x\right)\left(\sin\left(\gamma y\right)\cos\left(\beta z\right)+\cos\left(\gamma y\right)\sin\left(\beta z\right)\right)+C=0,$
P_II_ 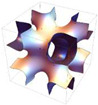	$0.5\left(\cos\left(2\alpha x\right)+\cos\left(2\beta z\right)+\cos\left(2\gamma y\right)\right)-\;\;\;\;-\left(\cos\left(\alpha x\right)\cos\left(\beta z\right)+\cos\left(\beta z\right)\cos\left(\gamma y\right)+\cos\left(\gamma y\right)\cos\left(\alpha x\right)\right)+C=0,$

**Table 4:** Regimes for studying of degradation rate.

Mode Name	Fluid	Temperature, °C
D–1	NaCl, 0.9%	37
D–2	NaCl, 0.9%	45

**Table 5:** The effect of the degradation mode on the elastic modulus (GPa) and compressive strength (MPa) of solid cylindrical specimens.

Days	Elastic Modulus, E (GPa)			Compression Strength, (MPa)		
	C–1	C–2	C–3	C–1	C–2	C–3
	no exposure					
–	7.86 ± 0.33 * (4.2%) **	5.55 ± 0.32 (5.8%)	5.05 ± 0.19 (3.8%)	84.56 ± 0.10 (0.1%)	83.85 ± 2.48 (3%)	78.14 ± 0.19 (0.2%)
	D–1 (37 °C)					
3	5.55 ± 0.97 (17.5%)	4.35 ± 0.44 (10.1%)	4.24 ± 0.33 (7.8%)	75.66 ± 3.60 (4.8%)	76.14 ± 3.68 (4.8%)	77.63 ± 2.68 (3.5%)
7	5.76 ± 1.06 (18.4%)	4.53 ± 0.51 (11.3%)	4.42 ± 0.28 (6.3%)	73.56 ± 4.10 (5.6%)	74.69 ± 3.72 (5%)	74.90 ± 3.13 (4.2%)
11	5.83 ± 1.34 (22.3%)	5.27 ± 0.22 (4.2%)	4.23 ± 0.44 (10.4%)	76.87 ± 2.58 (3.4%)	78.17 ± 1.30 (1.7%)	77.48 ± 1.49 (1.9%)
14	6.63 ± 0.50 (7.5%)	4.91 ± 0.21 (4.2%)	4.54 ± 0.29 (6.4%)	77.84 ± 1.30 (1.7%)	78.36 ± 1.32 (1.7%)	78.52 ± 1.26 (1.6%)
	D–2 (45 °C)					
3	4.70 ± 0.52 (11.1%)	3.61 ± 0.28 (7.8%)	3.93 ± 0.26 (6.6%)	64.36 ± 0.66 (1%)	61.02 ± 4.25 (7%)	67.92 ± 1.63 (2.4%)
7	4.26 ± 0.73 (17.1%)	3.76 ± 0.15 (4%)	3.44 ± 0.22 (6.4%)	60.08 ± 3.39 (5.6%)	59.61 ± 3.21 (5.4%)	62.87 ± 0.69 (1.1%)
11	4.07 ± 0.77 (18.9%)	3.64 ± 0.47 (12.9%)	3.53 ± 0.22 (6.2%)	59.03 ± 2.37 (4%)	61.94 ± 5.96 (9.2%)	60.49 ± 7.17 (11.9%)
14	4.16 ± 0.42 (10.1%)	3.43 ± 0.32 (9.3%)	3.44 ± 0.14 (4.1%)	58.32 ± 2.57 (4.4%)	57.02 ± 2.90 (5.1%)	58.81 ± 1.21 (2.1%)

* Standard deviation. ** Coefficient of variation (CV).

**Table 6:** Effective elastic moduli and compressive strength of structures with different orientation of printing layers.

Specimens	P–1	P–2
Elastic modulus, E (GPa)	5.28 ± 0.19 * (3.6%) **	5.55 ± 0.03 (0.5%)
Compression strength, (MPa)	67.03 ± 3.82 (5.7%)	75.18 ± 0.68 (0.9%)

* Standard deviation. ** Coefficient of variation (CV).

**Table 7:** The change in the mechanical characteristics of P–2 samples after two weeks of aging under different degradation regimes.

	No Exposure	D–1	D–2
Elastic modulus, GPa	5.55 ± 0.03 * (0.5%) **	5.52 ± 0.1 (1.8%)	5.37 ± 0.05 (0.9%)
Compressive strength (MPa)	75.18 ± 0.68 (0.9%)	72.57 ± 0.95 (1.3%)	65.18 ± 0.62 (1%)

* Standard deviation. ** Coefficient of variation (CV).

**Table 8:** Compressive strength (MPa) of lattice specimens under different degradation regimes.

Sample	Degradation Mode	No Exposure	Degradation Stage (Day)			
			3	7	11	14
DDM	D–1	15.67 ± 0.13 * (0.8%) **	17.12 ± 0.51 (3%)	16.18 ± 0.73 (4.5%)	16.98 ± 0.29 (1.7%)	16.93 ± 0.51 (3%)
	D–2		14.51 ± 0.12 (0.8%)	12.79 ± 1.14 (8.9%)	13.64 ± 0.60 (4.4%)	13.70 ± 0.64 (4.7%)
DP_II_M	D–1	15.48 ± 0.70 (4.5%)	16.21 ± 0.34 (2.1%)	15.38 ± 0.81 (5.3%)	15.07 ± 1.32 (8.8%)	14.21 ± 1.19 (8.4%)
	D–2		13.30 ± 1.34 (10.1%)	11.83 ± 0.78 (6.6%)	12.79 ± 0.51 (4%)	12.84 ± 0.45 (3.5%)

* Standard deviation. ** Coefficient of variation (CV).

**Table 9:** The morphometric characteristics of samples as a function of time and temperature of exposure in liquid medium.

	DP_II_M		DDM	
	No exposure			
Tb.BV, mm^3^	196.53		233.25	
SSA, mm^−1^	1.77		2.09	
Tb.Th., mm:	1.48 ± 0.88 (max 3.27)		0.90 ± 0.28 (max 1.63)	
	D–1			
	7 days	14 days	7 days	14 days
Tb.BV, mm^3^	193.25	177.16	177.86	174.00
SSA, mm^−1^	2.29	3.01	3.71	4.73
Tb.Th., mm:	1.34 ± 0.88 (max 3.19)	1.39 ± 1.02 (max 3.51)	0.85 ± 0.30 (max 1.5)	0.68 ± 0.46 (max 1.74)
	D–2			
	7 days	14 days	7 days	14 days
Tb.BV, mm^3^	194.47	186.02	192.16	178.52
SSA, mm^−1^	2.26	3.34	2.97	3.87
Tb. Th., mm:	1.37 ± 0.92 (max 3.21)	0.98 ± 0.68 (max 3.56)	0.64 ± 0.45 (max 1.62)	0.80 ± 0.38 (max 1.42)

**Figure A1:**
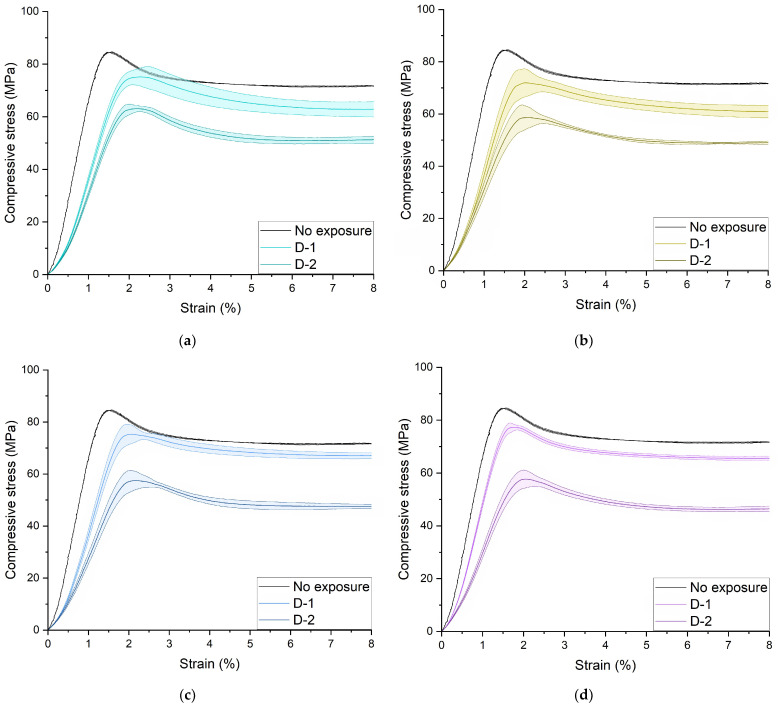
Averaged experimental stress–strain curves for C–1 specimens for degradation regimes D–1 and D–2 at different stages: (**a**) 3 days; (**b**) 7 days; (**c**) 11 days; (**d**) 14 days.

**Figure A2:**
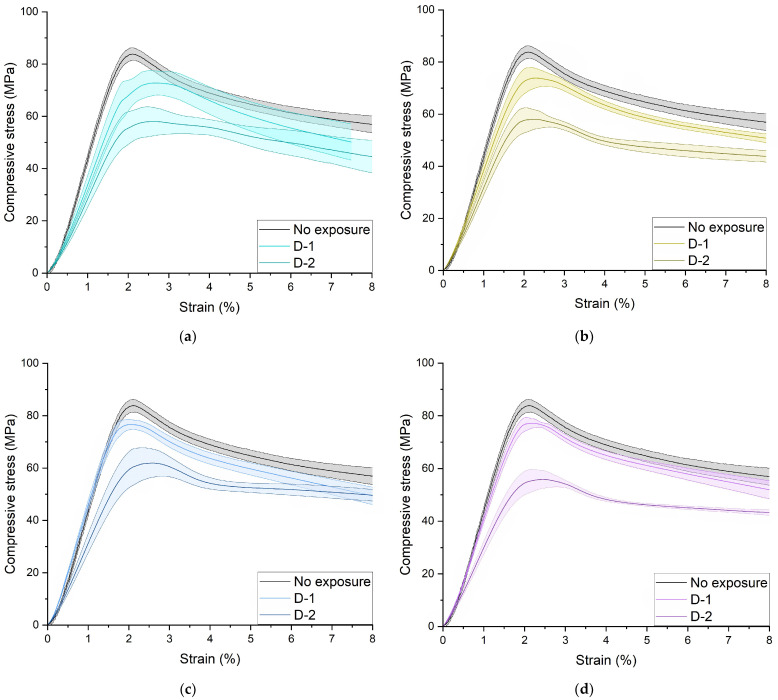
Averaged experimental stress–strain curves for C–2 specimens for degradation regimes D–1 and D–2 at different stages: (**a**) 3 days; (**b**) 7 days; (**c**) 11 days; (**d**) 14 days.

**Figure A3:**
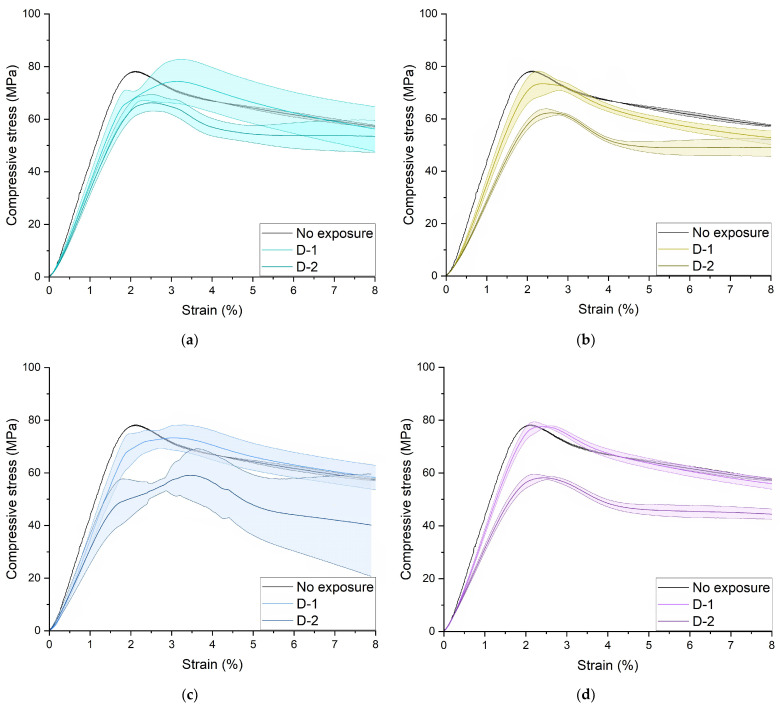
Averaged experimental stress–strain curves for C–3 specimens for degradation regimes D–1 and D–2 at different stages: (**a**) 3 days; (**b**) 7 days; (**c**) 11 days; (**d**) 14 days.

**Figure A4:**
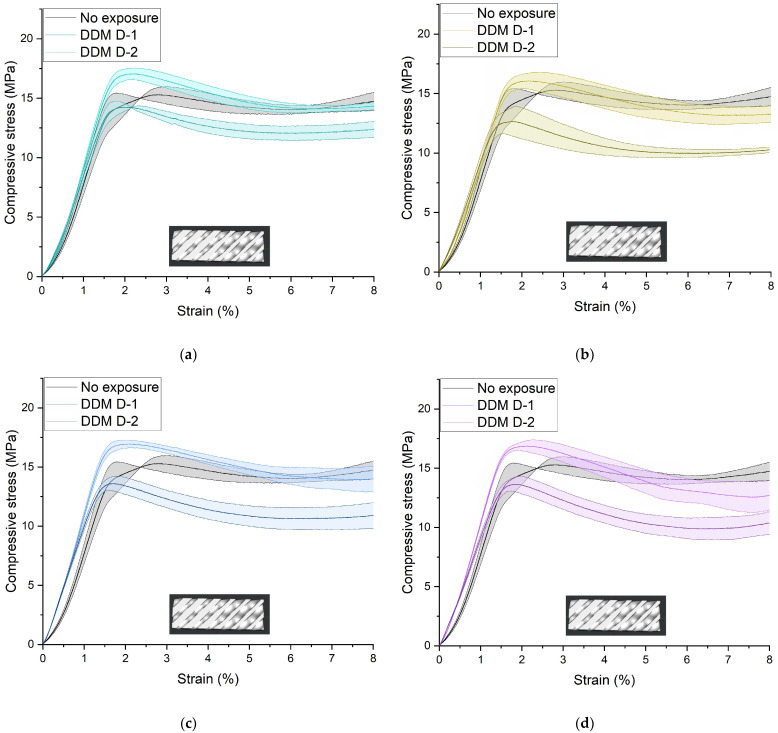
Averaged experimental stress–strain curves for DDM lattice structures for degradation regimes D–1 and D–2 at different stages: (**a**) 3 days; (**b**) 7 days; (**c**) 11 days; (**d**) 14 days.

**Figure A5:**
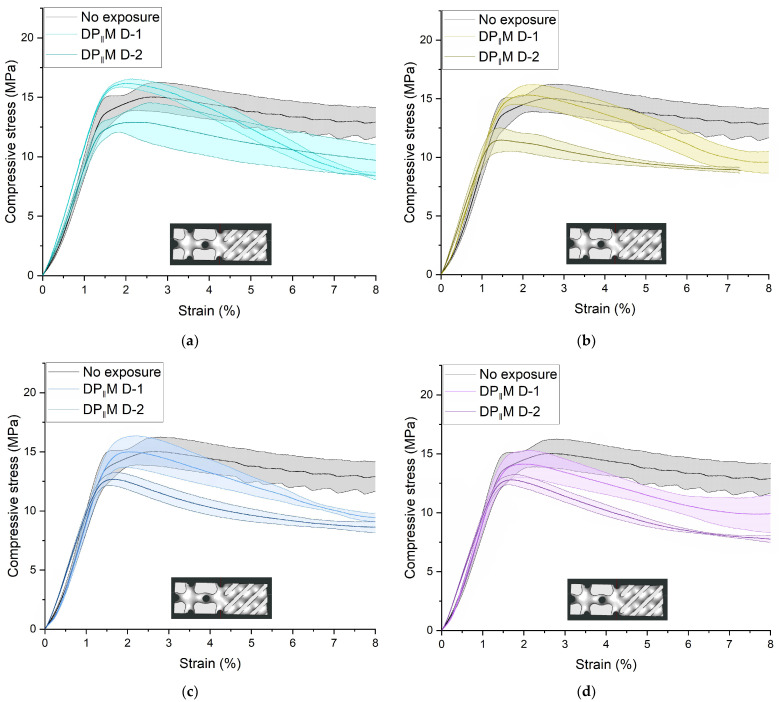
Averaged experimental stress–strain curves for DP_II_M lattice structures for degradation regimes D–1 and D–2 at different stages: (**a**) 3 days; (**b**) 7 days; (**c**) 11 days; (**d**) 14 days.

**Figure A6:**
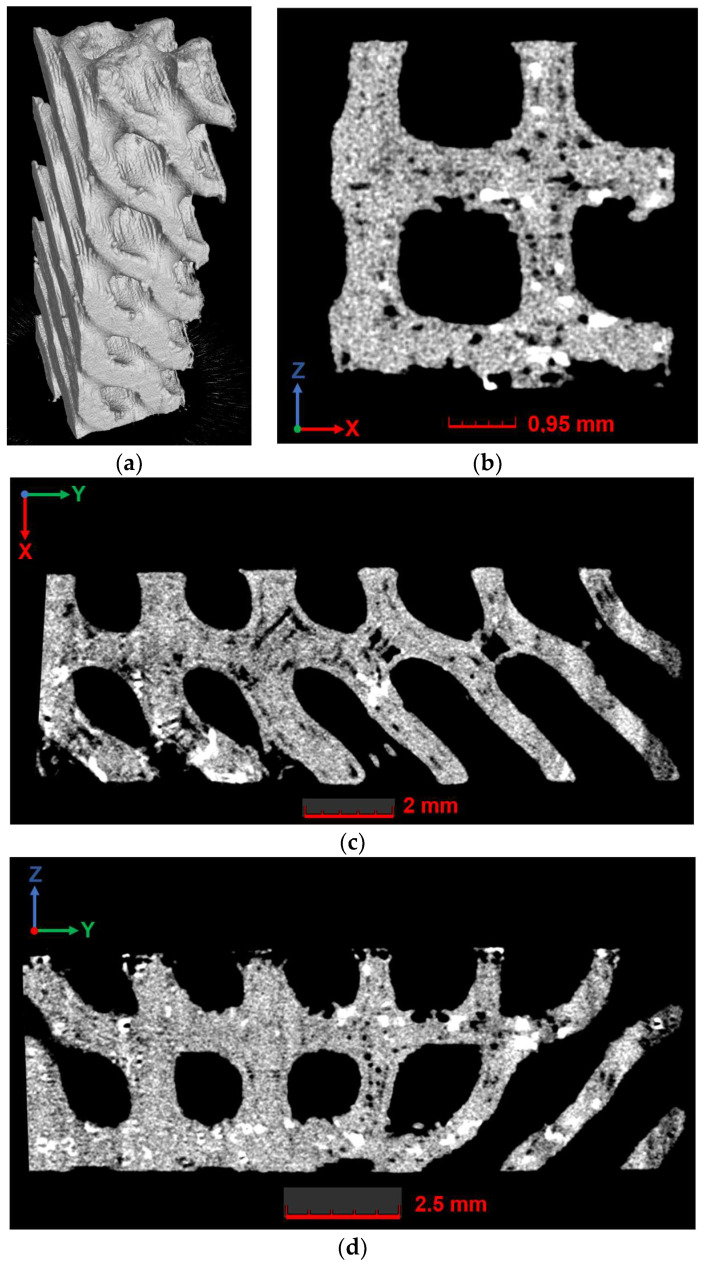
The results of micro-CT for the DDM sample with degradation in mode D–1 on day 14: (**a**) three-dimensional representation; (**b**) cross-section in the XZ plane; (**c**) cross-section in the XY plane; (**d**) cross-section in the YZ plane.

**Figure A7:**
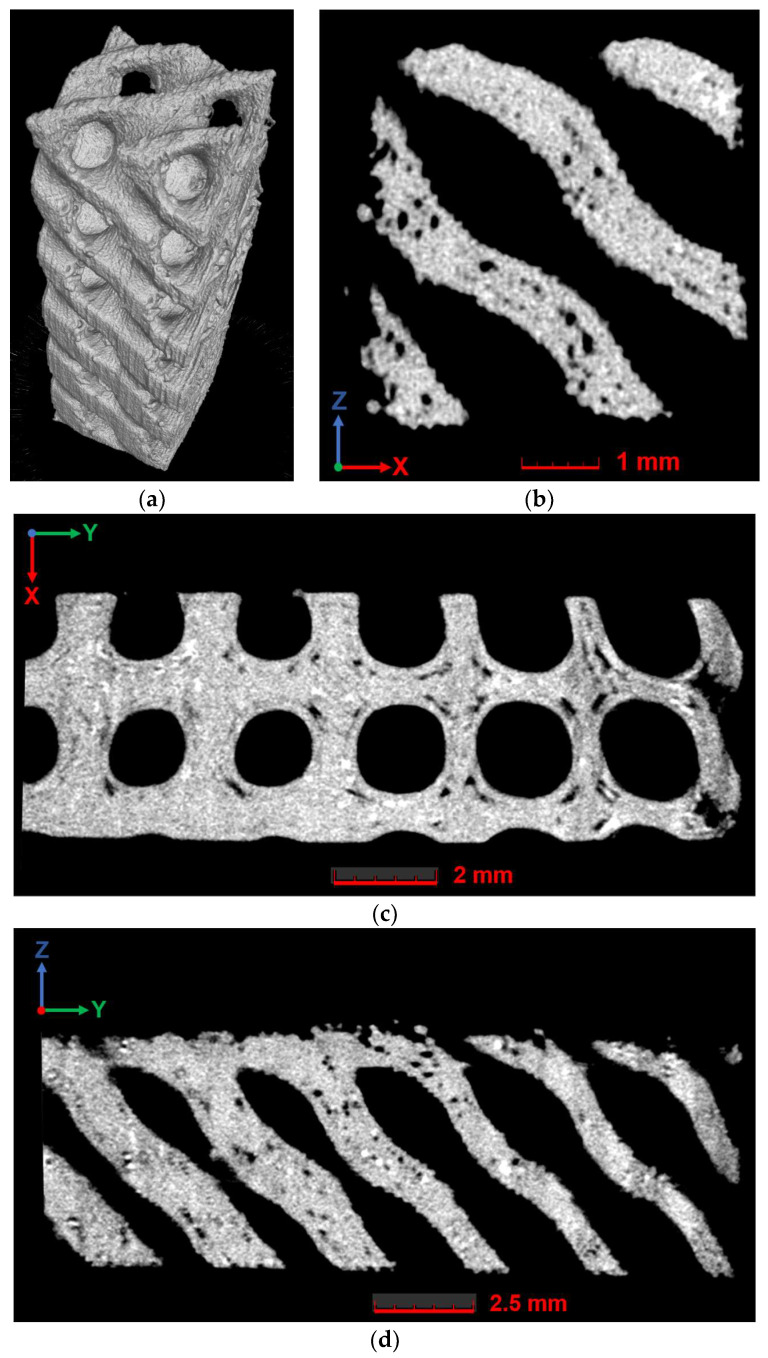
The results of micro-CT for the DDM sample with degradation in mode D–2 on day 14: (**a**) three-dimensional representation; (**b**) cross-section in the XZ plane; (**c**) cross-section in the XY plane; (**d**) cross-section in the YZ plane.

**Figure A8:**
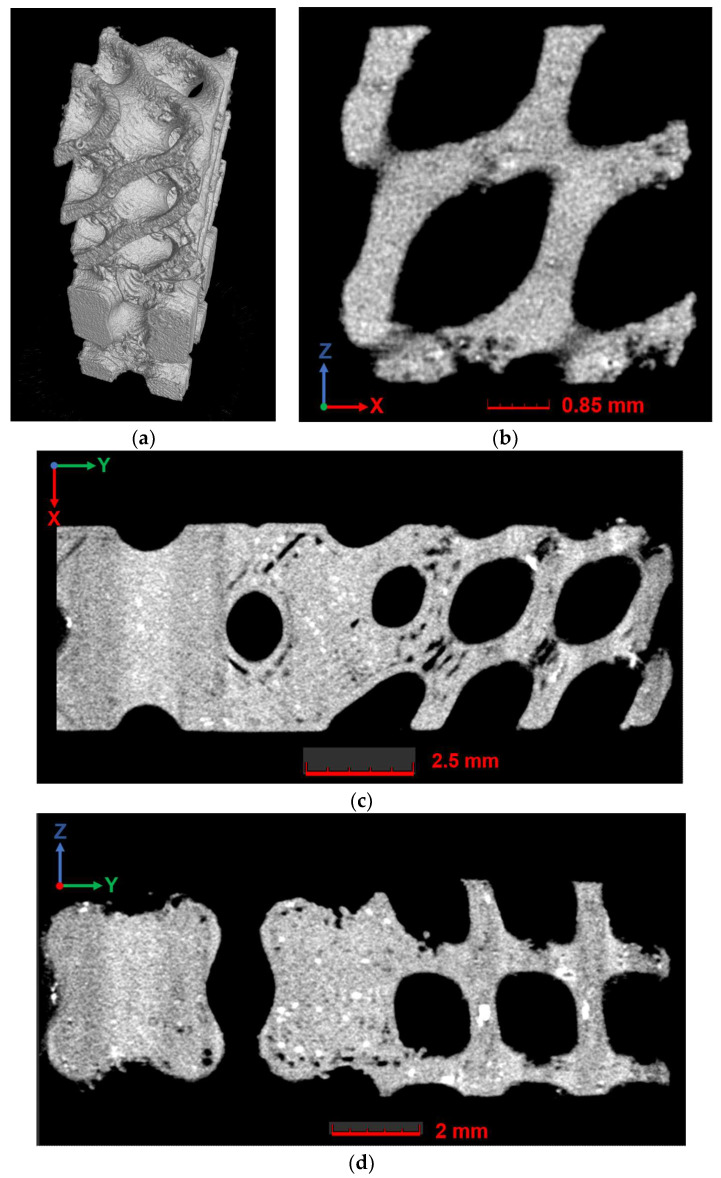
The results of micro-CT for the DP_II_M sample with degradation in mode D–2 on day 14: (**a**) three-dimensional representation; (**b**) cross-section in the XZ plane; (**c**) cross-section in the XY plane; (**d**) cross-section in the YZ plane.

## Data Availability

The data presented in this study are available on request from the corresponding author.

## Appendix A

For lattice specimens, the calculation of the compressive strength was performed using two methods: based on maximum engineering stresses and based on the relationship between the instantaneous elastic modulus and strain. The results of the calculations are summarized in Table A1.

**Table A1:** Comparison of calculated strength results obtained by different calculation methods.

Days	DDM		DP_II_M	
	Dependence of Instantaneous Elastic Modulus on Deformations	In Terms of Maximum Stress Values	Dependence of Instantaneous Elastic Modulus on Deformations	In Terms of Maximum Stress Values
	no exposure			
–	15.62 ± 0.15	15.67 ± 0.13	15.42 ± 0.70	15.48 ± 0.70
	D–1			
3	17.07 ± 0.53	17.12 ± 0.51	16.16 ± 0.36	16.21 ± 0.34
7	16.14 ± 0.74	16.18 ± 0.73	15.34 ± 0.80	15.38 ± 0.81
11	16.94 ± 0.29	16.98 ± 0.29	15.03 ± 1.31	15.07 ± 1.32
14	16.89 ± 0.52	16.93 ± 0.51	14.19 ± 1.20	14.21 ± 1.19
	D–2			
3	14.46 ± 0.13	14.51 ± 0.12	13.25 ± 1.32	13.30 ± 1.34
7	12.75 ± 1.13	12.79 ± 1.14	11.79 ± 0.78	11.83 ± 0.78
11	13.61 ± 0.61	13.64 ± 0.60	12.75 ± 0.51	12.79 ± 0.51
14	13.67 ± 0.63	13.70 ± 0.64	12.80 ± 0.46	12.84 ± 0.45

Since the discrepancy between the results of calculations obtained by the two methods does not exceed 1% on average, it was decided to henceforth calculate the ultimate strength based on maximum stresses.

## Appendix B

The mechanical performance was evaluated for solid specimens (C–1, C–2, C–3) and lattice structures (DDM and DP_II_M) from 5 trials for each type of specimen without environmental exposure and for each degradation step. The averaged results with confidence interval for each step are presented in Figure A1, Figure A2, Figure A3, Figure A4 and Figure A5.

## Appendix C

The effective elastic response to compression of lattice scaffolds was determined for control samples without any influence and for samples subjected to degradation under conditions of immersion in a liquid medium at elevated temperatures. The obtained results are summarized in Table A2.

**Table A2:** The compressive elastic modulus (GPa) of lattice specimens under different degradation regimes.

Days	Sample	
	DDM	DP_II_M
	no exposure	
–	1.23 ± 0.01	1.21 ± 0.07
	D–1	
3	1.27 ± 0.1	1.23 ± 0.01
7	1.25 ± 0.08	1.25 ± 0.08
11	1.27 ± 0.02	1.19 ± 0.09
14	1.26 ± 0.06	1.17 ± 0.07
	D–2	
3	1.08 ± 0.16	1.04 ± 0.06
7	1.05 ± 0.11	1.12 ± 0.18
11	1.08 ± 0.07	1.15 ± 0.14
14	1.03 ± 0.09	1.09 ± 0.07

The data for both the DDM structure and the DP_II_M structure are in good agreement with the results for solid samples considered in the main part of the work. Under D–1 conditions, at the considered time stages, insignificant changes in elastic moduli are observed, associated with the gradual absorption of moisture by the sample (which leads to an increase in properties) and the ongoing process of bulk degradation (elastic modulus gradually decrease). Under higher temperature conditions (D–2 mode), the process of bulk degradation predominates already at the initial stage, which leads to a gradual decrease in the elastic modulus. At the same time, the elastic modulus of the DP_II_M structure decrease faster than those of the DDM structure.

## Appendix D

The study of the internal structure of scaffold prototypes under various degradation regimes revealed an increase in the number of technical pores in the filament layers on the 14th day of exposure, regardless of the temperature mode. Below are images corresponding to the volumetric reconstruction of the sample and its individual cross-sections. For the DDM structure, results are presented for samples subjected to D–1 (Figure A6) and D–2 (Figure A7) modes.

For the DP_II_M structure (Figure A7), results are presented for sample subjected to D–2 mode (Figure A8).

For all the models considered (in the Results section and in this appendix), an increase in the proportion of technological pores in the structure is characteristic, observed in individual cross-sections. Compared to the samples of the “control group”, internal technological pores are more common in degraded structures. Moreover, erosion is observed not only in the internal part of the structure but also on the surface, which suggests the presence of surface degradation processes.
